# Diffusion-weighted imaging as a follow-up modality for evaluation of major salivary gland function in nasopharyngeal carcinoma patients: a preliminary study

**DOI:** 10.1007/s00066-020-01580-5

**Published:** 2020-02-05

**Authors:** Wen-jun Fan, Feng Teng, Yan-rong Luo, Wei Yu, Qian Zhang, Yi-ping Lu, Lin Ma

**Affiliations:** 1 Medical School of Chinese PLA, No. 28 Fuxing Road, 100853 Beijing, China; 2 Armed Police Forces Corps Hospital of Henan Province, No. 1 Kangfu Road, 450052 Zhengzhou, China; 3 Department of Radiation Oncology, China-Japan Friendship Hospital, No. 2 Yinghuayuan Dongjie, 100029 Beijing, China; 4 Department of Radiation Oncology, First Medical Center of Chinese PLA General Hospital, No. 28 Fuxing Road, 100853 Beijing, China

**Keywords:** Diffusion-weighted imaging, Radiotherapy, Xerostomia, Salivary gland dysfunction, Nasopharyngeal carcinoma

## Abstract

**Purpose:**

To investigate the value of diffusion-weighted imaging (DWI) in assessing dynamic changes of major salivary gland function during follow-up post radiotherapy (RT) in nasopharyngeal carcinoma (NPC) patients.

**Materials and methods:**

31 consecutive patients with pathologically confirmed NPC scheduled for RT underwent six routine follow-up MRI examinations including DWI sequence prior to (pre-RT) and 1, 3, 6, 9, and 12 months post RT. Mean apparent diffusion coefficient (ADC) values of bilateral parotid glands (PGs) and submandibular glands (SMGs) were measured. Objective measurement of salivary flow rate (SFR) under unstimulated (uSFR) and stimulated conditions (sSFR) as well as subjective xerostomia assessment according to a patient-rated questionnaire were conducted before each MRI. Variance analysis was used to evaluate dynamic changes of ADC, SFR and xerostomia questionnaire summary scores (XQ-sum) at different timepoints and the correlation between ADC and XQ-sum. Pearson’s correlation test was used to evaluate the correlations between pre- and post-RT changes of ADC (ΔADC) and SFR (ΔSFR) or mean RT dose.

**Results:**

At each timepoint, ADCs of PGs were significantly lower than of SMGs, uSFR was significantly lower than sSFR. For both PGs and SMGs, ADC_post-RT_ were all higher than ADC_pre-RT_, with significant differences. ADC_1m-post-RT_ initially increased and changed little to ADC_3m-post-RT_, ADC_6m-post-RT_, ADC_9m-post-RT_, and ADC_12m-post-RT_, then gradually declined over time. The dynamic change trends of SFR were negatively paralleled to those of ADC, while that of XQ-sum was similar. Dose–response relationships were detected between salivary gland mean RT dose and ΔADC. In PGs, negative correlations between ΔsSFR_9m-post-RT_ and ΔADC_9m-post-RT_, and ΔsSFR_12m-post-RT_ and ΔADC_12m-post-RT_ were detected. In SMGs, negative correlations between ΔsSFR_12m-post-RT_ and ΔADC_12m-post-RT_, and ΔuSFR_12m-post-RT_ and ΔADC_12m-post-RT_ were also detected. The ADCs of patients with severe subjective xerostomia were significantly higher, while patients with moderate subjective xerostomia presented a tendency toward higher ADCs compared to those with mild xerostomia from 6 to 12 months post RT.

**Conclusion:**

As part of routine follow-up MRI in NPC patients, DWI might be a promising modality for follow-up assessing the dynamic changes of major salivary gland function and might be more powerful in the late post-RT period.

## Introduction

Nasopharyngeal carcinoma (NPC) is one of the most common malignancies in southern China and southeast Asia, with an extremely high geographical incidence of about 25–30 per 100,000 persons per year [1, 2]. Radiotherapy (RT) alone or in combination with chemotherapy is the primary radical treatment for NPC patients and provides high locoregional control. Major salivary glands such as parotid glands (PGs) and submandibular glands (SMGs) are both highly radiosensitive and often involved in or adjacent to RT targets designed for NPC and apt to injury even after low-dose radiation [3]. Radiation-induced salivary gland injury and consequences such as xerostomia or dry mouth are probably the most common persistent oral sequelae for NPC patients who receive therapeutic doses of RT. Patients with xerostomia have considerable difficulties with chewing and swallowing and are impaired in speech and social interactions, which have further implications with respect to quality of life, especially in long-term survivors [4–6]. The probability and severity of xerostomia mostly depends on the dose distributions in PGs and SMGs [7]. Lower radiation doses lead to a better-maintained salivary flow after RT. Management of radiation-induced xerostomia by, e.g., pharmacological interventions (e.g., amifostine, pilocarpine) or submandibular gland transfer, appears to be beneficial with insufficient evidence. These treatments are not routinely recommended due to potential adverse effects of pharmacological agents and treatment delay of invasive submandibular gland transfer [8]. Therefore, the key lies in prevention by radiation dose reduction in major salivary glands.

During the past two decades, high-precision intensity-modulated radiation therapy (IMRT) technology with better conformity and dose homogeneity, such as helical tomotherapy (HT), has been increasingly applied for NPC treatment. With IMRT, substantial dose reductions could be achieved to major salivary glands, resulting in retention of salivary output and amelioration of xerostomia without compromising dose delivery to the tumor [9–11]. But despite the emergence of HT, the incidence of xerostomia 1 year and 2 years post RT in NPC patients is still at a high level. Based on this, long-term follow-up evaluation of the dynamic physiological changes of irradiated salivary glands would be important to better understand the mechanism of radiation-induced injury, which in turn aids the investigation of methods to mitigate symptoms of xerostomia such as optimization of treatment plans before implementing RT.

Traditionally, radiation-induced salivary gland dysfunction can be assessed subjectively according to a patient’s symptoms as well as objectively using quantified saliva production or excretion (scintigraphy and/or saliva flow) [12]. Salivary gland scintigraphy (SGS) has been shown to be reliable in evaluating salivary gland function [13, 14]. But the usefulness of SGS is limited by its low spatial resolution, the complex procedure, and its involvement of ionizing radiation. Salivary flow rate (SFR) measurement is the most commonly applied objective method in the clinic to quantify the severity of irradiated salivary gland dysfunction, but its results do not depict any morphological or physiological change of the irradiated salivary glands [12].

Diffusion-weighted imaging (DWI), which can assess and quantify the structural and pathophysiological changes by visualizing the random thermal motion of water molecule diffusion, has been widely used as one of the routine follow-up MR examinations of NPC patients for decades. In recent years, initial studies [15–19] of DWI in assessing radiation-induced salivary gland dysfunction have been documented and observed significant alterations of apparent diffusion coefficients (ADCs), the quantitative parameter of DWI, of major salivary glands pre and post RT in NPC patients. Several of these studies [17–19] further found a correlation between pre- and post-RT changes of ADC (ΔADC_post-pre_) and volume atrophy of PGs as well as ratios of salivary excretion fraction (rEF), the quantitative parameter of SGS. But until now, most of these initial studies have merely focused on the evaluation of PG dysfunction, whereas severity of xerostomia does not only attribute to the injury of PG but also SMG [20, 21]. Meanwhile, nearly all previous relative studies only compared ADCs of major salivary glands at one timepoint after RT with that before RT, continuous observations are scarce. And the correlations between changes in ADC and clinical parameters, such as subjective xerostomia degree and SFR, remain unclear. Besides, to the best of our knowledge, studies assessing salivary gland dysfunction with DWI in NPC patients treated with HT have not been documented. It would be interesting to investigate the radiation-induced physiological changes of irradiated PGs and SMGs at a lower radiation dose by using HT.

Therefore, the purpose of this preliminary study was to observe the dynamic changes in ADCs of both PGs and SMGs in NPC patients 1 year post HT, to analyze whether alterations of PG and/or SMG ADCs correlated with mean radiation dose and changes in SFR under stimulated/unstimulated conditions at six different follow-up timepoints, to further compare ADCs among patients with different subjective degrees of xerostomia.

## Materials and methods

### Patients

This prospective study registered with number ChiCTR1900024328 in the Chinese Clinical Trial Registry was approved by the research ethics board of the Chinese PLA General Hospital. Written informed consent was obtained from all eligible patients prior to participation.

From July 2017 to January 2018, 31 consecutive patients with pathologically confirmed NPC who were scheduled for bilateral neck HT with curative intent were prospectively recruited in this study. All patients had a good performance status (ECOG 0–1) and were scheduled to undergo MR evaluation and follow-ups at our hospital. None of them had received prior RT or surgery to the head and neck region, any previous salivary gland diseases such as Sjogren’s syndrome, any other medical causes of xerostomia, MRI contraindications, or any metal implants in the mouth. All patients underwent six MR examinations with an identical scan protocol each time, namely 2–3 weeks before RT (pre-HT) and 1, 3, 6, 9, and 12 months after completion of RT (1m-post-RT, 3m-post-RT, 6m-post-RT, 9m-post-RT, 12m-post-RT). Meanwhile, measurement of SFR as well as assessment of subjective degree of xerostomia were conducted an hour before each MR examination.

### Treatment

All patients were treated with induction chemotherapy followed by concurrent chemoradiotherapy. A two-cycle TP or TPF regimen was used in induction chemotherapy, i.e., docetaxel (T) 70 mg/m^2^ on day 1 and cisplatin (P) 40 mg/m^2^ on days 1 and 2; for patients with massive tumors at the primary site, 5‑fluorouracil (F) 700 mg/m^2^ on days 1–5 was added accordingly. Concomitant chemotherapy used docetaxel 70 mg/m^2^ or cisplatin 70 mg/m^2^ for three cycles every 21 days. RT was performed with HT (TomoTherapy; Accuray Inc., Sunnyvale, CA, USA) to the nasopharyngeal lesions, metastatic lymphadenopathy, and the neck lymphatic drainage areas. The total prescribed doses within the nasopharyngeal lesions and metastatic lymphadenopathy were 67.5 Gy with 2.25 Gy per fraction, while that within high-risk areas was 60 Gy with 2.0 Gy per fraction, and in low-risk areas 54 Gy with 1.8 Gy per fraction. The range of high-risk area and low-risk area was determined by the extent of nasopharyngeal lesions as well as the region of lymph node metastasis. RT was delivered once daily, five fractions per week, with a total of 30 fractions for six weeks. Treatment planning was optimized on the Pinnacle 3.8.0 treatment workstation (Philips Medical Systems, Fitchburg, WI, USA) by the reverse intensity-modulated planning system. All subjects were scheduled for a salivary gland-sparing technique to keep radiation doses to bilateral PGs and SMGs as low as possible on the promise of meeting the prescription dose coverage of 95% or more to the treatment volume. The mean radiation dose constraints for bilateral PGs were no more than 28 Gy, so do SMG-spared (ipsilateral level I b out of high-risk area). No salivary protectors or stimulants were allowed during therapy or the 1‑year study follow-up period. Details of plan design and dose–volume constraints for organs at risk referred to a previous article of our center [22].

### MRI protocol

All MRI scans were performed on a 3.0-T MR system with a maximum gradient capability of 23 mT/m (Signa HDx, GE Healthcare, Milwaukee, WI, USA). Patients were imaged in the supine position using a 16-channel neurovascular head and neck array coil. For morphologic evaluation, an axial T1-weighted spin-echo sequence (TR/TE, 428/11.6 ms) was performed using a matrix of 288 × 192, a field of view (FOV) of 180 × 240 mm, and a section thickness of 5 mm with an intersection gap of 1 mm. Then, fast spin-echo T2-weighted (TR/TE, 3000/59.5 ms) with fat suppression was obtained in the transverse plane with a matrix of 320 × 224, an FOV of 180 × 240 mm, and a section thickness of 5 mm with an intersection gap of 1 mm. This sequence was performed as a reference for the following DWI images to improve the delineation of both PGs and SMGs. The images encompassed the area from the skull base to the level of the glottis, including the full volume of PGs and SMGs.

Thereafter, an axial echoplanar DWI sequence was performed with a TR of 5000 ms, a minimum TE of 74.6 ms, a matrix of 128 × 128, and excitations. The other parameters, including FOV, section thickness, and intersection spacing, were identical to those used for T2-weighted imaging. The b values used were 0, 600 s/mm^2^. The motion-probing gradients were placed on the three orthogonal directions with the same strength. Eleven to thirteen sections were obtained, and the acquisition time of the DWI sequence was about 2 min.

### Image analysis

The data were digitally transferred from the MRI unit console to an independent Linux workstation with dedicated software (Advantage Workstation version 4.3, GE Healthcare). ADC maps were automatically constructed for DWI images by pixel-by-pixel calculation. ADC values were calculated using a mono-exponential model: *S*_*i*_ = *S*_0_ × exp(−*b*_*i*_ × ADC), where *S*_*i*_ is the signal intensity measured on the *i*-th *b* value image, *b*_*i*_ is the corresponding *b* value, and *S*_0_ indicates the exact (without noise) signal intensity for *b* = 0 s/mm^2^.

All the MR images were independently analyzed and measured by two radiologists with more than 8 years of experience in head and neck MR imaging, who were blinded to all clinical information. The regions of interest (ROIs) were manually drawn on the largest three contiguous slices of ADC images of bilateral PGs and SMGs to encompass as much of the gland parenchyma as possible in reference to T2-weighted images. The regions containing large vessels, such as the retromandibular vein and external carotid artery were excluded. The final ADC value of each salivary gland was defined as the mean value of three slices. The change rates of ADCs were calculated using the following equation:

$$\Delta \mathrm{ADC}_{(i)\mathrm{m}-\text{post-RT}}=(\mathrm{ADC}_{(i)\mathrm{m}-\text{post-RT}}-\mathrm{ADC}_{\text{pre-RT}})/\mathrm{ADC}_{\text{pre-RT}}\times 100\%$$

Where (*i*)m-post-RT represents the exact month after completion of RT, ΔADC_(__*i*__)m-post-RT_ is the change rate of ADC value at (*i*)m-post-RT compared with pre-RT, and ADC_pre-RT_ is the ADC value before RT.

The final results were recorded as the mean value of two observers’ measurements. ADC values of both PGs and SMGs were repeatedly measured by one radiologist at an interval of 4 weeks to evaluate the intra-observer reproducibility.

### Whole-mouth SFR measurement

Whole-mouth SFR measurement, one kind of sialometry, directly measures the function of salivary glands. In our study, whole-mouth salivary output measurement was conducted using the spitting method. Patients were instructed to spit their saliva in a graded tube for 5 min, both at rest and upon stimulation by dipping citric acid of 2% concentration on the tongue tip with a cotton bud once every 20 s. The collected saliva volume in the two conditions was recorded and then used to calculate SFR under unstimulated (uSFR) as well as SFR under stimulated conditions (sSFR) as well as their change rates using the following equation:

$$\Delta \mathrm{SFR}_{(i)\mathrm{m}-\text{post-RT}}=(\mathrm{SFR}_{(i)\mathrm{m}-\text{post-RT}}-\mathrm{SFR}_{\text{pre-RT}})/\mathrm{SFR}_{\text{pre-RT}}\times 100\%$$

Where ΔSFR_(__*i*__)m-post-RT_ is the change rate of SFR at (*i*)m-post-RT compared with pre-RT, and SFR_pre-RT_ is SFR before RT.

Patients were asked not to eat or drink for at least 1 h before the examination.

### Assessment of the degree of xerostomia

Before each SFR measurement, patients were asked to complete a modified xerostomia-specific questionnaire which has been validated previously [23]. Briefly, the questionnaire consists of five items regarding dryness when eating, speaking, swallowing, and chewing, and five relating to dryness while at rest. Each item is rated on a four-point ordinal scale from 0 to 3, with higher scores indicating more severe xerostomia. The score of each item is summed up to produce a summary score ranging from 0 to 30. The severity of xerostomia is classified by the summary score (XQ-sum): mild xerostomia, XQ-sum ≤10 points; moderate xerostomia, 10 < XQ-sum ≤ 20 points; severe xerostomia, XQ-sum > 20 points.

### Statistical analysis

Statistical analysis was performed with SPSS 24.0 Software (IBM Corp., Armonk, NY, USA). Descriptive statistics were used to analyze patients’ demographic data and clinical characteristics. Numerical data were presented as the mean ± standard deviation. Kolmogorov–Smirnov’s test was used to determine whether the parameters were normally distributed. Paired two-tailed Student’s *t-*tests were used to compared mean radiation doses to PGs and SMGs, ADCs of PGs and SMGs, and SFR under different conditions at each timepoint. The dynamic changes of ADCs and SFR, and the differences in ADCs among different degrees of xerostomia were analyzed by repeated measures analysis or one-way analysis of variance with the Bonferroni post-test. Correlations between ΔADC and ΔSFR and mean radiation doses were investigated using Pearson’s correlation test. The intra- and inter-observer reproducibility of ADC values were analyzed using the intraclass correlation coefficient (ICC). Two-sided *P-*values less than 0.05 were considered as statistically significant.

## Results

**Fig. 1:**
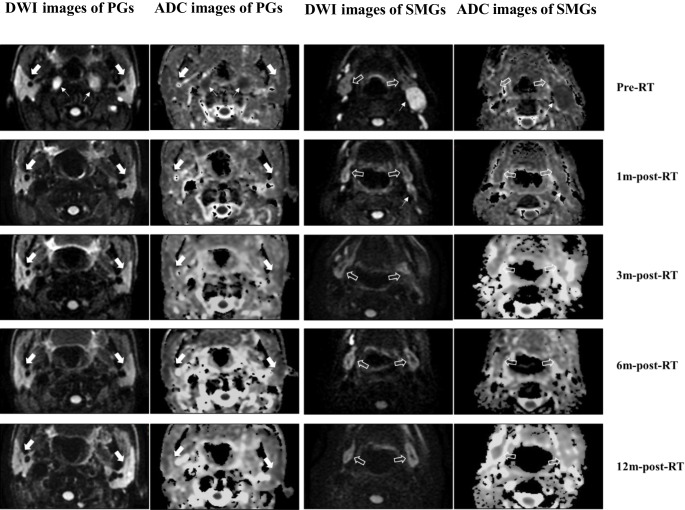
Diffusion weighted imaging (DWI) and apparent diffusion coefficient (ADC) images of bilateral parotid glands (*PGs*; *white solid arrow*) and submandibular glands (*SMGs*; *white hollow arrow*) of a 49-year-old male NPC patient at different timepoints pre and post RT. For both PGs and SMGs, ADC images illustrated a relatively slightly higher signal at 1m-post-RT and 3m-post-RT compared to that of pre-RT, with a gradually decreasing signal at 6m-post-RT and 12m-post-RT. Meanwhile, the metastatic lymph nodes (*white arrow*) apparently disappeared after 1m-post RT

All subjects successfully underwent the whole therapy and follow-up MR examinations. No measurement was excluded because of insufficient quality. The pre- and post-RT DWI and ADC images of bilateral PGs and SMGs of one representative subject are shown in Fig. 1. Excellent reproducibility of the measurement of ADC for both PGs and SMGs was achieved, with the inter-observer ICC at 0.92 (*P* < 0.001) and the intra-observer ICC at 0.94 (*P* < 0.001). The main characteristics of subjects and tumor are presented in Table 1.

**Table 1:** Clinical characteristics of patients and tumors

Characteristic	Value
Patients (male/female)	31 (27/4)
Age (years)	
Mean (range)	48.6 (18–67)
T stage	
T1	4 (12.9%)
T2	15 (48.4%)
T3	6 (19.4%)
T4	6 (19.4%)
N stage	
N1	2 (6.5%)
N2	23 (74.2%)
N3	6 (19.4%)
Clinical stage	
Stage II	1 (3.2%)
Stage III	20 (64.5%)
Stage IVA	10 (32.3%)
Mean radiation dose (Gy)	
PG‑R	30.62 ± 2.91 (27.10–40.18)
PG‑L	29.80 ± 2.47 (26.70–37.79)
SMG‑R	46.64 ± 16.61 (24.47–65.59)
SMG‑L	41.27 ± 16.86 (22.38–66.35)

*PG‑L* left parotid gland, *PG‑R* right parotid gland, *SMG‑L* left submandibular gland, *SMG‑R* right submandibular gland

### Comparison of PGs and SMGs

There was no difference in mean radiation dose to bilateral PGs or SMGs, whereas the mean radiation dose to SMGs was significantly higher than to PGs (*P* < 0.001), as shown in Table 1. At each timepoint, ADCs of bilateral PGs were similar to each other, the same was true for bilateral SMGs, while ADCs of PGs were significantly lower than those of SMGs (*P* < 0.05), as shown in Table 2.

**Table 2:** Summary of measurement characteristics

Mean values ± SD	ADC (× 10^−3^mm^2^/s)		ΔADC (%)		SFR (ml/min)		ΔSFR (%)		XQ-sum (scores)
	PGs	SMGs	PGs	SMGs	Unstimulated	Stimulated	Unstimulated	Stimulated	
Pre-RT	1.08 ± 0.11	1.38 ± 0.14^a^	–	–	0.32 ± 0.25	0.87 ± 0.70^b^	–	–	0.46 ± 0.21
1m-post-RT	1.61 ± 0.19	1.79 ± 0.25^a^	48.87 ± 18.45	29.19 ± 13.23	0.13 ± 0.14	0.27 ± 0.25^b^	−55.94 ± 24.55	−49.07 ± 34.27	12.97 ± 5.24
3m-post-RT	1.63 ± 0.22	1.83 ± 0.26^a^	50.64 ± 20.80	32.03 ± 16.82	0.10 ± 0.09	0.31 ± 0.27^b^	−56.79 ± 20.17	−47.76 ± 27.91	12.65 ± 4.35
6m-post-RT	1.53 ± 0.21	1.71 ± 0.25^a^	41.67 ± 20.50	23.65 ± 14.92	0.14 ± 0.13	0.40 ± 0.34^b^	−46.60 ± 23.34	−32.24 ± 28.37	10.65 ± 4.12
9m-post-RT	1.41 ± 0.19	1.61 ± 0.27^a^	31.24 ± 20.04	16.67 ± 16.43	0.16 ± 0.15	0.47 ± 0.40^b^	−30.74 ± 23.86	−29.48 ± 29.62	9.52 ± 4.49
12m-post-RT	1.31 ± 0.19	1.52 ± 0.26^a^	21.72 ± 19.83	9.70 ± 16.87	0.23 ± 0.18	0.62 ± 0.44^b^	−11.61 ± 10.71	−12.08 ± 12.37	6.45 ± 3.02

*RT* radiation therapy, *pre-RT* 2–3 weeks before RT, *(i)m-post-RT* the *i*th month after completion of RT, *PGs* parotid glands, *SMGs* submandibular glands, *ADC* apparent diffusion coefficient, *ΔADC* (ADC_post-HT_ − ADC_pre-HT_)/ADC_pre-HT_, *SFR* saliva flow rate, *ΔSFR* (SFR_post-HT_ − SFR_pre-HT_)/SFR_pre-HT_, *XQ-sum* summary scores of xerostomia questionnaire

^a^Denotes a significant difference between SMGs and PGs (*P* < 0.001)

^b^Denotes a significant difference between SFR under stimulated and unstimulated conditions (*P* ≤ 0.001)

### Dynamic changes of ADC at different follow-up timepoints

**Fig. 2:**
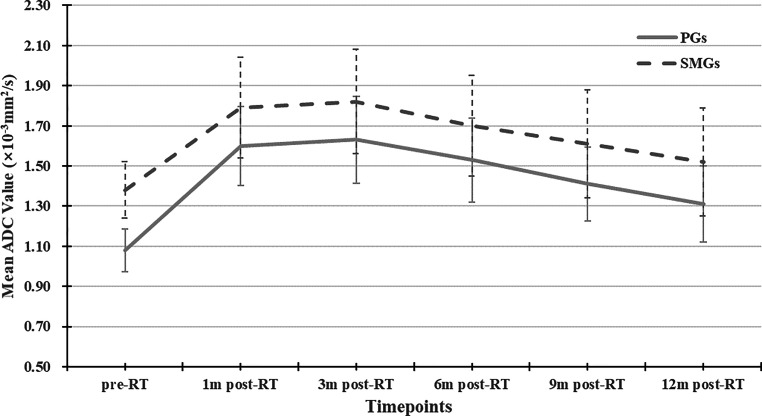
Dynamic changes of ADC of both parotid glands (*PGs*) and submandibular glands (*SMGs*). Line graphs indicate a similar change trend over time for both PGs and SMGs, i.e., ADC increased significantly 1m-post-RT, followed by no obvious change until 3m-post-RT, and then gradually declined

The change trends over time of the ADC of PGs and SMGs were similar, as shown in Fig. 2. With either PGs or SMGs, ADC_post-RT_ were all higher than ADC_pre-RT_, with significant differences (*P* ≤ 0.01); ADC_1m-post-RT_ and ADC_3m-post-RT_ were nearly equal (*P* = 1.000); ADC_6m-post-RT_, ADC_9m-post-RT_, ADC_12m-post-RT_ became lower and lower over time (*P* < 0.05). But for SMGs, the statistical differences between ADC_6m-post-RT_ and ADC_9m-post-RT_, and ADC_9m-post-RT_ and ADC_12m-post-RT_ lacked significance (*P* = 0.457 and 0.493, respectively).

### Dynamic changes of SFR at different follow-up timepoints

**Fig. 3:**
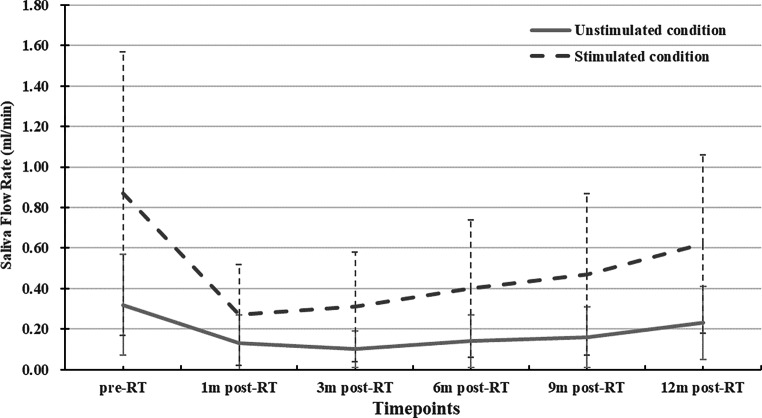
Dynamic changes of SFR under unstimulated (uSFR) and stimulated conditions (sSFR). Line graphs indicate a similar change trend over time for both uSFR and sSFR, i.e., SFR decreased significantly 1m-post-RT, followed by no obvious change until 3m-post-RT, and then gradually increased

At each timepoint, sSFR was higher than uSFR (*P* < 0.001 for all), as shown in Table 2. For both sSFR and uSFR, SFR_1m-post-RT_ apparently declined from SFR_pre-RT_, changed little to SFR_3m-post-RT_, then gradually increased over time, as shown in Fig. 3. SFR_post-RT_ values were all lower than SFR_pre-RT_, with the exception that the difference between SFR_12m-post-RT_ and SFR_pre-RT_ was not statistically significant (*P* = 0.737 for sSFR, *P* = 0.738 for uSFR). The differences in SFR between two continuous timepoints post RT were not significant (*P* > 0.05 for all). But there were significant differences between SFR_12m-post-RT_ and SFR_3m-post-RT_ of sSFR (*P* = 0.022), and SFR_12m-post-RT_ and SFR_6m-post-RT_ of uSFR (*P* = 0.013).

### Correlation of ΔADC with RT dose as well as ΔSFR

As shown in Table 3, linear correlations were found between mean radiation dose to PGs and ΔADC_post-pre_, and the dose–response relationship was seen at each follow-up timepoint post RT. Linear correlations were also found between mean radiation dose to SMGs and ΔADC_6m-post-RT_, ΔADC_9m-post-RT_, and ΔADC_12m-post-RT_. There was a significant negative correlation between ΔsSFR_9m-post-RT_ and ΔADC_9m-post-RT_ (*r* = −0.369, *P* = 0.040), and ΔsSFR_12m-post-RT_ and ΔADC_12m-post-RT_ (*r* = −0.411, *P* = 0.021) of PGs, between ΔsSFR_12m-post-RT_ and ΔADC_12m-post-HT_ (*r* = −0.417, *P* = 0.020), and ΔuSFR_12m-post-RT_ and ΔADC_12m-post-RT_ (*r* = −0.392, *P* = 0.029) of SMGs.

**Table 3:** Correlation between ΔADC (%) and mean radiation dose and ΔSFR (%)

Correlation coefficient (*r*)		Mean radiation dose	ΔuSFR					ΔsSFR				
			1m-post-RT	3m-post-RT	6m-post-RT	9m-post-RT	12m-post-RT	1m-post-RT	3m-post-RT	6m-post-RT	9m-post-RT	12m-post-RT
ΔADC-PGs	1m-post-RT	0.467^a^	−0.042	–	–	–	–	−0.285	–	–	–	–
	3m-post-RT	0.420^a^	–	−0.149	–	–	–	–	−0.213	–	–	–
	6m-post-RT	0.446^a^	–	–	−0.330	–	–	–	–	−0.289	–	–
	9m-post-RT	0.409^a^	–	–	–	−0.291	–	–	–	–	*−0.369*^*a*^	–
	12m-post-RT	0.371^a^	–	–	–	–	−0.338	–	–	–	–	*−0.411*^*a*^
ΔADC-SMGs	1m-post-RT	0.143	−0.087	–	–	–	–	−0.157	–	–	–	–
	3m-post-RT	0.239	–	−0.042	–	–	–	–	−0.182	–	–	–
	6m-post-RT	0.376^a^	–	–	−0.061	–	–	–	–	−0.302	–	–
	9m-post-RT	0.405^a^	–	–	–	−0.322	–	–	–	–	−0.322	–
	12m-post-RT	0.447^a^	–	–	–	–	*−0.392*^*a*^	–	–	–	–	*−0.417*^*a*^

*ΔADC-PGs* change in apparent diffusion coefficient of parotid glands, *ΔADC-SMGs* ΔADC of submandibular glands, *ΔuSFR* change in salivary flow rate under unstimulated conditions (at rest), *ΔsSFR* ΔSFR under stimulated condition (citric acid of 2% concentration)

^a^Denotes a significant correlation between ΔADC and mean radiation dose (*P* < 0.001)

^b^Denotes a significant correlation between ΔADC and ΔSFR (*P* < 0.05)

### Relationship between ADC and subjective degree of xerostomia

**Fig. 4:**
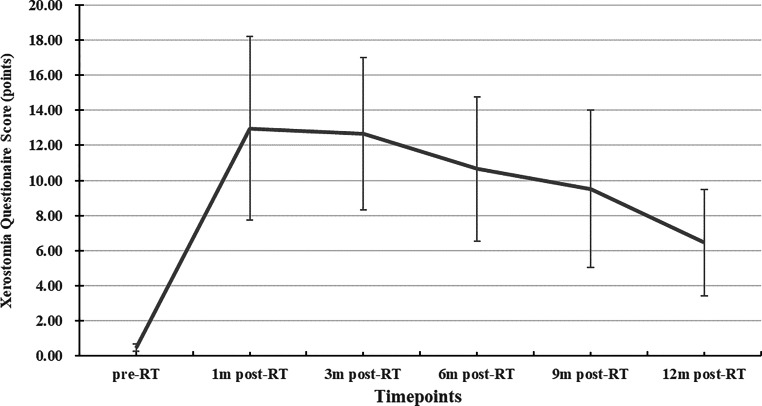
Dynamic changes of xerostomia questionnaire summary score (XQ-sum). Line graph indicating change trend over time of XQ-sum shows an initial increase 1m-post-RT with subsequently minor change until 3m-post-RT and then a gradual decrease from 3m-post-RT to 12m-post-RT

**Fig. 5:**
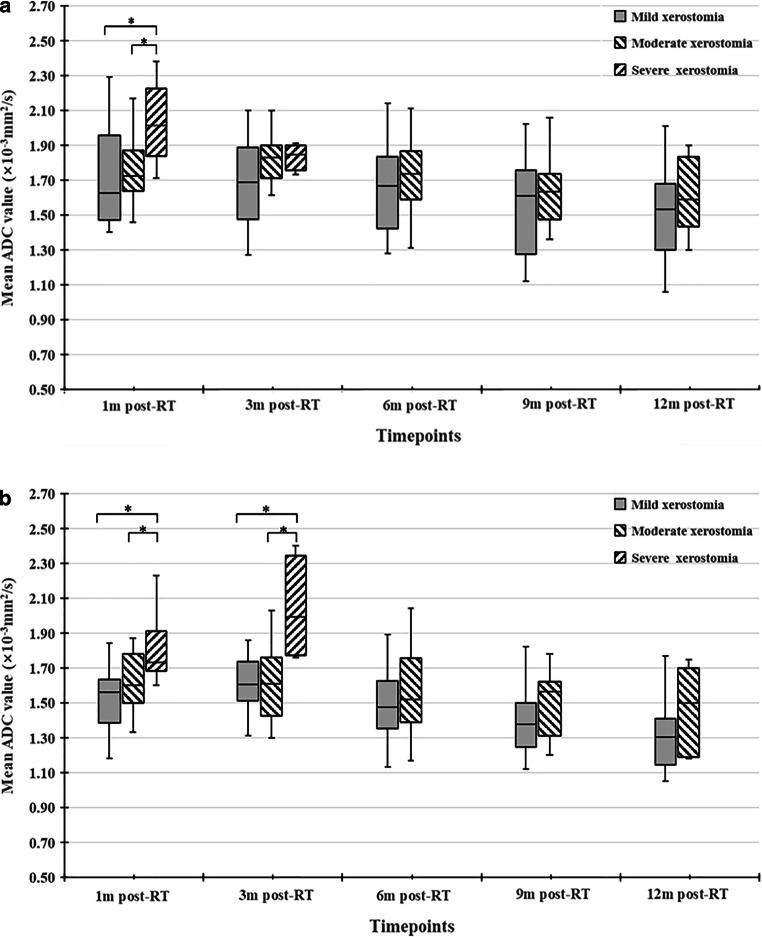
Comparison of ADC of PGs (**a**) and SMGs (**b**) among patients with different degrees of xerostomia classified by XQ-sum (mild xerostomia, XQ-sum ≤10 points; moderate xerostomia, 10 < XQ-sum ≤ 20 points; severe xerostomia, XQ-sum > 20 points). Boxplots among different degrees of xerostomia show higher ADCs in patients with severe xerostomia in the early post-RT period (1m-post-RT) and a tendency toward higher ADCs in patients with moderate xerostomia compared to those with mild xerostomia in the late post-RT period (from 6m-post-RT to 12m-post-RT) for both PGs and SMGs, despite a lack of statistical difference (*denote a significant difference compared to severe xerostomia; i.e., *P* < 0.05)

As shown in Fig. 4, the overall change trend post RT of XQ-sum was in line with that of ADC. From 3m-post-RT, XQ-sum gradually decreased over time, with XQ-sum_12m-post-RT_ apparently lower. Classified by XQ-sum, the percentage of patients with mild, moderate, and severe xerostomia at 1m-post-RT was 35.5%, 48.4%, and 16.1%, respectively, while at 12m-post-RT, the percentage of patients with mild xerostomia increased to 90.3%, moderate xerostomia declined to 9.7%, and no patient had severe xerostomia. With further analysis, ADC_1m-post-RT_ and ADC_3m-post-RT_ of PGs and ADC_1m-post-RT_ of SMGs in patients with mild and moderate xerostomia were significantly higher than in those with severe xerostomia (*P* < 0.05 for both). Compared to patients with mild xerostomia, ADC_1m-post-RT_ and ADC_3m-post-RT_ of PGs and SMGs in patients with moderate xerostomia showed no significant differences, but ADC_6m-post-RT_, ADC_9m-post-RT_, and ADC_12m-post-RT_ were all higher, albeit lacking statistically significant differences (*P* > 0.05 for all), as shown in Fig. 5.

## Discussion

Xerostomia is defined by the Common Toxicity Criteria of Adverse Events (CTCAEs) as a disorder characterized by reduced salivary flow in the oral cavity. PGs and SMGs are the main sources of saliva flow. Stimulated salivary production is largely derived from PGs (approximately 60% of total saliva in stimulated conditions), while resting or unstimulated saliva is mostly produced by SMGs (nearly 90% of total saliva in unstimulated conditions) [24]. Radiation-induced xerostomia is mostly attributed to injury of PGs and SMGs. To accurately evaluate post-RT changes of salivary gland function or xerostomia, different assessment methods have been reported in the literature [12, 24]. Our study using DWI to assess dynamic changes of salivary gland function over time in 1‑year post-RT and particularly to assess PGs and SMGs separately, found a strong correlation between pre- and post-RT changes of ADC and those of SFR under unstimulated/stimulated conditions as well as with the degree of subjective xerostomia. To the best of our knowledge, this is the first follow-up research so far to explore the correlations of DWI parameters with clinical parameters containing objective SFR measurement and subjective degree of xerostomia simultaneously.

PGs and SMGs are composed of acinar cells, which produce saliva. Animal studies [25, 26] found that both irradiated PGs and SMGs were characterized by acinar cell loss or atrophy, interstitial fibrosis, and so forth. In our study, at each timepoint either pre or post RT, ADCs of SMGs were significantly higher than of PGs. This finding is in accordance with previous studies [18, 19] and it is thought to reflect the much higher proportional amount of extracellular water of SMG. Compared to ADC_pre-RT_, ADC_post-RT_ rise significantly. This rise is thought to reflect the increased water diffusivity in injured salivary glands as a result of acinar cellular loss. After 3‑months post RT, ADC became lower and lower over time. The drop of ADC is thought to reflect the decreased water diffusivity owing to the increase of acinar cell number, which indicates functional recovery of salivary glands. A previous rat model study [25] observed an initial drop of acinar cell number of PGs followed by an increase in the late post-irradiation period (120–240 days).

Regarding the dosage effect, the relationship between radiation dose absorbed by salivary glands and ΔADC_post-pre_ has previously been investigated. In their series of 34 NPC patients, Marzi et al. [27] found no significant correlation between the RT dose and ADC changes of PGs from measurement timepoints at half way through and at the end of RT. Loimu et al. [18] found a dose–response correlation between radiation dose to PGs as well as SMGs and ΔADC_post-pre_ from measurement timepoints prior to RT onset and at a mean of 6 months post RT. Our study further showed significantly positive correlations between radiation dose and ΔADC_post-pre_ at five timepoints from 1 month to 12 months post RT of PGs and ΔADC_post-pre_ at 6, 9, and 12 months post RT of SMGs, which indicates a dose-dependent loss of acinar cells and a continuous dose–response effect over time.

SFR measurement remains the benchmark for assessment of salivary gland function. It directly measures the function of salivary glands and can be classified into a whole-mouth output measurement and a selective salivary gland output measurement. The former measurement is relatively fast, noninvasive, and easily achieved, which renders it more commonly used in clinics. Our study employed the whole-mouth output measurement to acquire SFR under unstimulated and stimulated conditions. In our study, sSFR was higher than uSFR at each timepoint. The overall change trend of SFR was negatively paralleled to that of ADC; SFR declined in the first 3 months post RT, then gradually increased over time. This finding corresponds with the study of Gupta [14], who found a maximal decrease in rEF at 3m-post-RT with then functional recovery over time in a cohort of 41 head and neck cancer patients by using quantitative SGS to assess PG function. The difference between SFR_12m-post-RT_ and SFR_pre-RT_ was not statistically significant, but the differences between SFR_12m-post-RT_ and SFR_3m-post-RT_ of sSFR and SFR_12m-post-RT_ and SFR_6m-post-RT_ of uSFR were significant. The results indicated that both uSFR and sSFR could recover nearly to the pre-RT level 1‑year post RT, but the time to begin recovery of sSFR was ahead of uSFR.

One unique feature of our study is the correlation between ΔADC and ΔSFR under different conditions at different follow-up timepoints, which, to the best of our knowledge, has not been investigated before. In the study, significant negative correlations were found between ΔSFR_9m-post-RT_ and ΔADC_9m-post-RT_, and ΔSFR_12m-post-RT_ and ΔADC_12m-post-RT_ of PGs, as well as ΔSFR_12m-post-RT_ and ΔADC_12m-post-RT_ of SMGs in the acid-stimulated condition and ΔSFR_12m-post-RT_ and ΔADC_12m-post-RT_ of SMGs in the resting condition. The correlations were in accordance with physiological function of saliva production as mentioned above: both PGs and SMGs produce the majority of saliva during stimulation, while SMGs alone produce the majority of saliva while at rest. Furthermore, the correlations manifest that DWI can not only evaluate salivary gland dysfunction post RT separating PGs from SMGs, but also be quite suitable for assessing it in the late post-RT period.

Evaluation of the degree of xerostomia is also widely used in assessing salivary gland dysfunction clinically, and can be classified into operator-rated outcomes according to classification systems such as CTCAE, the Radiation Therapy Oncology Group (RTOG) toxicity criteria, and patient-rated outcomes using questionnaires [28, 29]. In a cohort of 23 NPC patients receiving IMRT, Zhang et al. [19] found no differences in ADCs between patients with grade 1 and grade 2 xerostomia classified according to the RTOG toxicity criteria. But recent studies [30] have found that operator-rated outcomes might underestimate the actual degree of xerostomia and patient-rated outcomes are uniquely able to provide actual information. Another unique feature of our study is comparison of ADCs among different degrees of xerostomia graded by patient-rated outcomes at different follow-up timepoints. The questionnaire used in the study was the modified edition of a xerostomia-specific questionnaire which has been validated previously [23, 31]. The classification of xerostomia severity based on XQ-sum correlated to the grading system of dry mouth of CTCAE version 5.0 on the whole, with mild, moderate, and severe xerostomia equal to grade 1, 2, and 3 dry mouth by CTCAE version 5.0, respectively. The overall change trend of XQ summary scores was in keeping with that of ADC, which indicates the consistence of ADC changes and subjective xerostomia over time after RT. In early post-RT period, ADC of both PGs and SMGs in patients with mild xerostomia and those with moderate xerostomia showed no obvious differences, while those with severe xerostomia were much lower. Meanwhile, patients with moderate subjective xerostomia presented the tendency of higher ADCs compared to those with mild xerostomia in the late post-RT period, but statistically significant differences were lacking, which might be due to the small size of our study. Based on this, we consider that although DWI is not sufficient to distinguish mild from moderate subjective xerostomia in the early post-RT period, it can fully distinguish severe subjective xerostomia and has the potential to differentiate mild from moderate subjective xerostomia in the late post-RT period.

It is estimated that at least 1‑year of follow-up is required to determine the effect of RT on xerostomia [32]. Thus, the follow-up period in our present study may be considered as sufficient to provide adequate information in terms of xerostomia following RT. Additionally, the inter- and intra-observer reproducibility of ADC of both PGs and SMGs was excellent, indicating high reliability of the measurement of DWI parameters of PGs as well as SMGs.

This preliminary study has some limitations. First, despite being larger than previous DWI studies on evaluation of radiation-induced salivary gland dysfunction, the sample size was still small, especially for patients with severe subjective xerostomia. Many more samples with different degrees of xerostomia should be included in future studies. Second, our study employed three continuous slices covering the largest area of both PGs and SMGs to represent the entire salivary glands for analysis rather than including all slices. In consideration of intra-tissue heterogeneity, earlier research [33] reported regional differences in radio-sensitivity inside the salivary glands, so sampling errors could not be avoided. Regional analysis of ADCs in salivary glands should be considered in further research. Third, it has been verified that ADC is not only affected by water molecular diffusion but also by microvascular perfusion [34]. The advanced intravoxel incoherent motion DWI (IVIM-DWI) technique can separate contributions of diffusion and perfusion, which makes it more informative than DWI for assessing radiation-induced salivary gland damage. Despite this, some current deficiencies such as a much longer acquisition time and a complex post-processing procedure restrict clinical applications of IVIM. Conversely, with the advantages of easily achieved fast acquisition (usually no more than 2 min) and simple post-processing operation, DWI is much more convenient for patients. Thus, we consider that it is more easily achievable and suitable to use DWI as the follow-up method for evaluation of salivary gland function after RT. Last but not least, the impact of chemotherapy on salivary gland function is currently unclear and it is still an open question owing to various antineoplastic drugs, doses, and cycles. Strigari et al. [31] found that concomitant chemotherapy had no impact on xerostomia by univariate analysis in a cohort of 63 head and neck cancer patients (44 with NPC). To the best of our knowledge, there are currently no studies on the effects of chemotherapy on ADC of salivary glands present. Preliminary results of our center showed no significant differences of ADC of both PGs and SMGs pre and post induction chemotherapy. But more samples are needed to verify the results.

## Conclusion

This study showed that for both PGs and SMGs, during 1 year of follow-up post RT, the dynamic change trends of ADC were negatively paralleled to those of objective measurement (SFR under unstimulated and stimulated conditions) and in line with those of patient-reported subjective degree of xerostomia (XQ scores), indicating that DWI might be a useful modality for follow-up assessing radiation-induced physiological and functional changes of major salivary glands in NPC patients. Moreover, in the late post-RT period, the dynamic change rates of ADC were negatively correlated with those of SFR, and patients with moderate subjective xerostomia presented a tendency toward higher ADCs compared to those with mild xerostomia, suggesting that follow-up DWI evaluation might be a powerful evaluation tool in this period.
